# Beyond type 2 diabetes, obesity and hypertension: an axis including sleep apnea, left ventricular hypertrophy, endothelial dysfunction, and aortic stiffness among Mexican Americans in Starr County, Texas

**DOI:** 10.1186/s12933-016-0405-6

**Published:** 2016-06-08

**Authors:** Craig L. Hanis, Susan Redline, Brian E. Cade, Graeme I. Bell, Nancy J. Cox, Jennifer E. Below, Eric L. Brown, David Aguilar

**Affiliations:** Human Genetics Center, School of Public Health, The University of Texas Health Science Center at Houston, Houston, TX 77225 USA; Division of Sleep and Circadian Disorders, Brigham and Women’s Hospital, Harvard Medical School, Boston, MA 02115 USA; Beth Israel Hospital, Boston, MA 02215 USA; Departments of Medicine and Human Genetics, The University of Chicago, Chicago, IL 60637 USA; Vanderbilt Genetics Institute, Vanderbilt University School of Medicine, Nashville, TN 37232 USA; Center for Infectious Disease, School of Public Health, The University of Texas Health Science Center at Houston, Houston, TX 77225 USA; Cardiology, Baylor College of Medicine, Houston, TX 77030 USA

**Keywords:** Type 2 diabetes, Obesity, Hypertension, Aortic stiffness, Endothelial function, Sleep apnea, Left ventricular mass, Prevalence, Hispanic, Mexican American

## Abstract

**Background:**

There is an increasing appreciation for a series of less traditional risk factors that should not be ignored when considering type 2 diabetes, obesity, hypertension, and cardiovascular disease. These include aortic stiffness, cardiac structure, impaired endothelial function and obstructive sleep apnea. They are associated to varying degrees with each disease categorization and with each other. It is not clear whether they represent additional complications, concomitants or antecedents of disease. Starr County, Texas, with its predominantly Mexican American population has been shown previously to bear a disproportionate burden of the major disease categories, but little is known about the distribution of these less traditional factors.

**Methods:**

Type 2 diabetes, obesity and hypertension frequencies were determined through a systematic survey of Starr County conducted from 2002 to 2006. Individuals from this examination and an enriched set with type 2 diabetes were re-examined from 2010 to 2014 including assessment of cardiac structure, sleep apnea, endothelial function and aortic stiffness. Individual and combined frequencies of these inter-related (i.e., axis) conditions were estimated and associations evaluated.

**Results:**

Household screening of 5230 individuals aged 20 years and above followed by direct physical assessment of 1610 identified 23.7 % of men and 26.7 % of women with type 2 diabetes, 46.2 and 49.5 % of men and women, respectively with obesity and 32.1 and 32.4 % with hypertension. Evaluation of pulse wave velocity, left ventricular mass, endothelial function and sleep apnea identified 22.3, 12.7, 48.6 and 45.2 % of men as having “at risk” values for each condition, respectively. Corresponding numbers in women were 16.0, 17.9, 23.6 and 28.8 %. Cumulatively, 88 % of the population has one or more of these while 50 % have three or more.

**Conclusions:**

The full axis of conditions is high among Mexican Americans in Starr County, Texas. Individual and joint patterns suggest a genesis well before overt disease. Whether they are all mediated by common underlying factors or whether there exist multiple mechanisms remains to be seen.

**Electronic supplementary material:**

The online version of this article (doi:10.1186/s12933-016-0405-6) contains supplementary material, which is available to authorized users.

## Background

Accumulating evidence establishes a series of less traditional risk factors not to be ignored in considerations of diabetes, obesity, hypertension, and cardiovascular disease. These factors include aortic stiffness, cardiac structure, impaired endothelial function, obstructive sleep apnea, coronary artery calcification, carotid intima-media thickness and ankle/brachial index. They vary in difficulty in their implementation and appear to be associated to varying degrees with each disease categorization and with each other [1–5], but it is not clear whether they represent additional complications or concomitants of disease. There are few reports of their combined impact in the general population and even less in the Hispanic population. We report here the individual age- and sex-specific impacts of type 2 diabetes, obesity, hypertension, aortic stiffness, left ventricular hypertrophy, impaired endothelial function and sleep apnea among Mexican Americans in Starr County, Texas. We also report their associations with each other, with prediabetes and normal glycemia and their cumulative impact. These results, coupled with those now reported from the Hispanic Community Health Study/Study of Latinos for the traditional factors [6, 7] and sleep disordered breathing [8], clearly demonstrate an underappreciated and under-targeted burden of disease.

As we age, arterial stiffness increases [9] leading to increased central pressure and left ventricular load that likely contribute to left ventricular hypertrophy [10] and may independently predict cardiovascular events [11]. These processes seem to be exacerbated in type 2 diabetes [12]. Left ventricular hypertrophy [13], in particular, is a consequence of hypertension and independently predicts coronary heart disease and heart failure [14]. It shows associations with diabetes [14], sleep apnea [15] and pulse wave velocity [16]. Changes in the regulatory capacity of the endothelium can also lead to increased aortic stiffness [17]. Arterial stiffness has been reported to be associated with obstructive sleep apnea [1, 3, 5, 18].

Impaired endothelial function has been demonstrated in individuals with prediabetes and diabetes [19, 20]. Worsening endothelial function appears to be linearly associated with dysglycemia even in the absence of overt diabetes [19] and is “ubiquitous” at all levels of insulin resistance and diabetes [21]. Furthermore, endothelial dysfunction is a common concomitant of obstructive sleep apnea [22, 23]. Among the largest and earliest efforts to understand the epidemiology of obstructive sleep apnea was the Sleep Heart Health Study established in 1994 [15, 24]. It and other studies make it clear that sleep apnea is intimately intertwined with an axis of non-traditional risk factors. It is associated with type 2 diabetes [3, 25], hypertension, cardiac morphology and cardiovascular disease [1, 17], obesity [5, 8] and all-cause mortality [26].

These non-traditional risk factors provide key physiologic targets where understanding their biological underpinnings could have substantial impact on developing strategies to slow the progression and prevent several chronic conditions. Recognition of their contribution is a relatively recent phenomenon and additional population data are needed across most ethnic groups. While each factor could be reported in isolation, it is important that their cumulative role and relationships be reported at a higher level. Here we report the impact of type 2 diabetes, obesity, hypertension, aortic stiffness, left ventricular mass, reactive hyperemia and sleep apnea among Mexican Americans obtained from systematic evaluation of the population of Starr County, Texas. The overall burden and relationships among these factors point to a staggering burden of chronic disease with underlying commonalities and unique relationships among them.

## Methods

### Study design

**Fig. 1:**
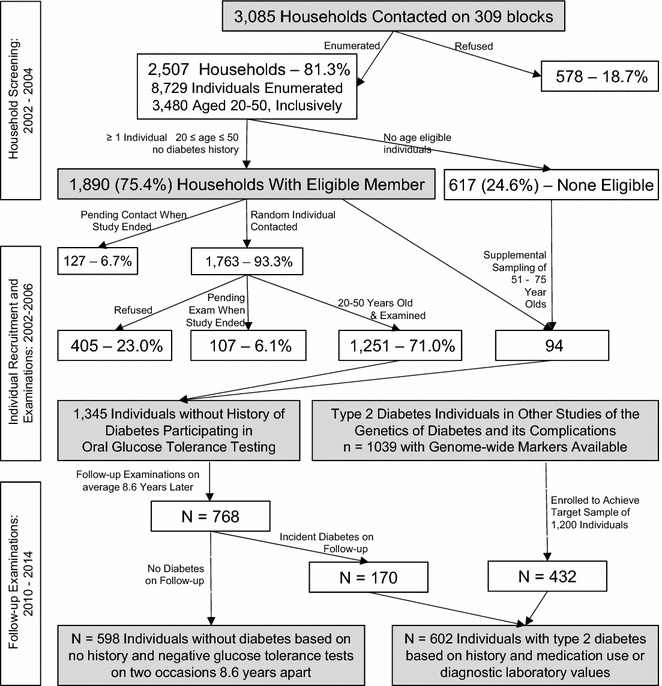
Flow diagram summarizing the household survey and two examination rounds of selected individuals

The data presented were generated in three phases; an enumeration of households, detailed examination of selected individuals, and follow-up examinations. A flow chart of the sampling is presented in Fig. 1. From 2002 to 2004 we performed a systematic enumeration of 2507 households from 309 blocks selected randomly from the major population centers of Starr County. This led to the identification of 8729 individuals (14.9 % of the estimated 2004 population of Starr County) largely representative of the age and sex distribution of the overall population. Diabetes status by history was determined for all but one individual.

From each household, one individual aged 20–50 years and not previously diagnosed with diabetes was randomly selected for a physical evaluation following an overnight fast that included medical and demographic history, anthropometry, EKG and blood pressure measurement and an oral glucose tolerance test using a 75 g glucose load with sampling at 0, 30, 60, 90 and 120 min post-load. A total of 1251 individuals were examined. An additional 94 individuals over the age of 50 years without a previous diagnosis of diabetes were also examined to give estimates of the undetected load of abnormal glucose tolerance among older individuals bringing the total examined with this protocol to 1345. These examinations occurred from 2002 to 2006. Glucose levels were determined at our field site, in duplicate, using an YSI 2300 STAT Plus Glucose & Lactate Analyzer (YSI Life Sciences, Yellow Springs, Ohio). Blood pressure was measured three times following a 5 min period of sitting rest using a Hawksley random zero sphygmomanometer (Hawksley and Sons, Lancing, UK). The average systolic and diastolic pressures from the second and third measurements were used as an individual’s pressures.

From the household enumeration, 265 individuals with a history of diabetes and currently taking glucose lowering medications were enrolled and examined in an ongoing study of the genetics of type 2 diabetes and its complications. There were also 50 newly diagnosed cases of diabetes based on the oral glucose tolerance testing that were rolled into these genetic studies. These examinations shared protocols with the exceptions that no glucose tolerance tests were performed and sensitive eye examinations were added for classification of diabetic retinopathy [27].

Individuals participating in the oral glucose tolerance tests (n = 1345) and all those with type 2 diabetes participating in the genetics of diabetes studies (n = 1039) formed the pool of subjects eligible for a follow-up examination from December 2010 to January 2014. All were classified as Mexican American. These follow-up examinations were expanded in scope. The oral glucose tolerance was modified to add 10 and 20 min sampling points for those without diabetes. Echocardiography, evaluation of aortic stiffness, and assessment of reactive hyperemia and sleep apnea were also added. A target sample size of 1200 for these follow-up examinations was set with the intent of sampling equal numbers of those with and without diabetes. Sampling from the 1345 having an earlier oral glucose tolerance test continued until nearly 600 individuals remaining free of diabetes were examined. This sampling also identified 170 incident cases of type 2 diabetes from this group. To complete the targeted 600 individuals with type 2 diabetes, we examined 432 individuals with diabetes from our genetic studies. The final sample with follow-up examinations consisted of 598 individuals without diabetes and 602 individuals with type 2 diabetes (Fig. 1).

Standard 2D and Doppler echocardiographic measures were obtained using an Acuson Cypress (Siemens Medical Solutions, Mountain View, CA) and protocols reported previously [28]. As a measure of aortic stiffness, pulse wave velocity in meters per second (PWV) was determined from the common carotid to the femoral artery using the validated SphygmoCor CPV System (AtCor Medical, Sydney, Australia) [29] according to manufacturer recommendations. Transit distance for calculation of pulse wave velocity was determined by subtracting the distance from the carotid location to the sternal notch from the distance between the sternal notch and the femoral measurement site. Reactive hyperemia was measured as a surrogate for endothelial function using an EndoPat 2000 (Itamar Medical, Caesarea, Israel) to measure digital peripheral arterial tonometry changes following 5 min of brachial occlusion with a standard blood pressure cuff according to manufacturer recommendations. This method has been shown to be reproducible [30], less prone to operator variability [31] and highly correlated with brachial flow-mediated dilation [32].

An overnight, in-home sleep evaluation was performed using the WatchPat 200 monitor (Itamar Medical, Caesarea, Israel) based on digital peripheral arterial tonometry which has been shown to correlate well with polysomnography [33]. Sleep recordings were manually reviewed to ensure consistent identification of sleep, wake and artifact. The primary metric was the algorithm derived estimated apnea hypopnea index (AHI; number of respiratory disturbances per hour of estimated sleep). Blood pressure was measured three times following a 5 min sitting rest using an automated device (Critikon Dinamap, Tampa, FL) with the average of the second and third values used as the final measures.

### Disease and non-traditional risk factor definitions

Previously identified diabetes was based on a positive response to being asked if a health care professional had ever told the individual that they had diabetes. Individuals undergoing a physical evaluation were classified with diabetes based on a fasting glucose of 126 mg/dl or greater or a 2 h post-load glucose of 200 or greater or a previous diagnosis of diabetes and current use of glucose lowering medications according to current guidelines [34]. We used the same guidelines to classify those with impaired fasting glucose (100–125 mg/dl) or impaired glucose tolerance (2 h glucose of 140–199 mg/dl) as having prediabetes. In our most recent examination, all individuals were measured for HbA1c (Siemens DCA Vantage Analyzer point of care device, Malvern, PA) allowing additional classification of diabetes (HbA1c ≥ 6.5 %) or prediabetes (5.7 ≤ HbA1c ≤ 6.4 %) [34].

Overweight was defined as a body mass index (BMI, weight in kilograms divided by the square of height in meters) of 25 or more, but less than 30 while obesity was defined as a BMI of 30 or more [35]. Hypertension was classified as a systolic blood pressure (average of the second and third measures) of 140 mmHg or greater or a diastolic pressure of 90 or greater or current use of antihypertensive medications [36]. Individuals were classified with significant aortic stiffness based on a pulse wave velocity of 12 or greater [37]. A digital pulse reactive hyperemia index (RHI) of less than 1.67 was taken as evidence of a significant deficit according to manufacturer recommendations and as used in other settings [38]. Left ventricular (LV) mass was calculated from 2D measures and indexed to height in meters raised to the 2.7 power as detailed in Lang et al. [13] and rounded to the nearest whole. Mild, moderate and severe LV mass abnormalities were classified using sex-specific reference values (45–51, 52–58, 59+ g/m^2.7^, respectively for women and 49–55, 56–63 and 64+ g/m^2.7^, respective for men) from [13]. Owing to an equipment failure that led to several months without an echocardiograph, LV mass was obtained on only 733 subjects. Mild, moderate and severe sleep apnea were defined based on an AHI of 5, 15 and 30 or more, respectively [8]. Individuals reporting regular or intermittent use of a CPAP device were also classified as having severe sleep apnea. Among those with diabetes were a number of individuals that were older and frailer. These individuals were examined either in their homes or nursing facilities. As a result, their examinations included only a subset of the protocols that did not require the equipment in our field office.

### Statistical analysis

Frequencies of diabetes, prediabetes, overweight, obesity and hypertension come from the household survey coupled with the concurrent individual examinations (Fig. 1). Because the sampling involved those with and without diabetes separately, the final frequencies represent weighted frequencies reflective of the population distribution of those with and without diabetes. Sampling in the 2010–2014 follow-up examinations results in a representative sample of those without diabetes and a representative sample with diabetes, but the overall sample is no longer representative of the frequencies in the total population. We employ a similar weighting strategy as above accounting for the expected distribution of diabetes in the population for generating overall frequencies and show in figures and supplemental tables the impact of diabetes on all of these conditions.

Data are generally presented as frequencies stratified by sex, age group or disease classifications. Differences in frequencies among men and women are tested with the Mantel-Haenzel Chi square controlling for the effects of age groupings [39]. To examine the relationships between the multiple factors, a series of logistic regressions [40] were performed. Because near complete data were available for age, sex, diabetes, obesity and hypertension, these factors were always included in the logistic models except when they were being treated as the independent variable. Thus, the significance of any factor was determined in the context of adjustment for age, sex, diabetes, obesity and hypertension. This strategy maximized the sample size available for each analysis. When a factor was the dependent variable, each was dichotomized as follows: diabetes—yes or no; obesity—BMI ≥ 30 or not; LV mass—moderate and severe versus not; sleep apnea—AHI ≥ 30 or not. When analyzed as an independent variable, the full groupings were used.

## Results

Tables 1 and 2 detail the frequencies by age and sex of the full axis of traditional and non-traditional risk factors. In Table 1, age- and sex-specific prevalences of the traditional factors, type 2 diabetes, obesity and hypertension, are documented while Table 2 tabulates the less traditional factors of aortic stiffness, LV mass, reactive hyperemia and sleep apnea. Previously diagnosed diabetes comes from the 5230 individuals aged 20 and above in the representative household survey of the Starr County Mexican American population. An additional 3498 individuals were less than age 20 with diabetes reported for only five of them. Of the 648 individuals classified as having diabetes in the household enumerations, only one was consistent with type 1 diabetes based on an age at diagnosis under the age of 20, continuous insulin use since diagnosis and normal weight. All others were considered to have type 2 diabetes. No significant differences between men and women were seen for any of the diabetes related classifications. While the overall proportions of men and women with a BMI of 25 or greater were similar, men were significantly more often overweight and significantly less often obese than women after controlling for age group. Men were also significantly more often hypertensive than women, but only moderately so.

**Table 1:** Population frequencies of type 2 diabetes, obesity and hypertension by age and sex among Mexican Americans in Starr County, Texas

Age group	Sampling (n)		Type 2 diabetes				Overweight and obesity^d^		Hypertension^c^
	Survey	OGTT	Previously identified %^a^	Newly identified %^b^	Total diabetes %	Pre- diabetes %^b^	25 ≤ BMI < 30 %	BMI ≥ 30 %	SBP ≥ 140 or DBP ≥ 90 or current meds
Men									
20–29	585	141	1.9	2.8	4.7	22.3	37.6	38.0	5.7
30–39	519	118	4.8	9.7	14.5	27.4	39.3	44.9	13.9
40–49	405	103	13.1	11.8	24.9	32.9	37.4	50.3	25.6
50–59	351	22	18.2	14.9	33.1	44.6	38.0	45.3	44.4
60–69	254	12	22.8	19.3	42.1	38.6	29.5	51.9	70.5
70+	208	12	24.5	25.2	49.7	44.0	37.6	55.3	79.5
Totals	2322	408	11.8^c^	11.9^c^	23.7^c^	32.8^c^	37.0^c^	46.2^c^	32.1^c^
Women									
20–29	707	263	1.4	1.9	3.3	28.5	31.9	41.1	2.7
30–39	615	310	3.4	7.5	10.9	39.1	32.4	47.4	5.3
40–49	565	279	10.3	10.0	20.2	40.5	29.8	57.3	21.7
50–59	434	38	24.9	9.9	34.8	35.6	30.3	59.6	50.6
60–69	298	32	32.9	25.2	58.1	16.3	35.6	45.0	71.2
70+	289	13	29.8	32.4	62.2	21.6	30.9	46.9	84.8
Totals	2908	935	14.4^c^	12.3^c^	26.7^c^	31.9^c^	31.5^c^	49.5^c^	32.4^c^
p value			0.062	0.594	0.276	0.807	0.000	0.018	0.046
♂vs♀ and OR^e^			0.85	1.05	1.19	1.01	1.28	0.88	1.16

^a^Rates for previously identified diabetes come from the representative household survey including 5230 individuals aged 20 and above

^b^Based on oral glucose tolerance tests on 1345 individuals having no prior history of diabetes and then weighted to what would be seen in the total population

^c^Population frequency among those 20 and above adjusted to the 2010 Census by the direct method

^d^Weighted frequencies of overweight or obesity among those with normal glucose tolerance, pre-diabetes and type 2 diabetes based on the 1609 individuals from the household survey participating in full examinations

^e^p-value based on Mantel–Haenszel Chi square testing for difference by sex after controlling for age effects. OR is the Mantel–Haenszel adjusted odds ratio

**Table 2:** Aortic stiffness, LV mass, endothelial dysfunction and sleep apnea by age and sex among Mexican Americans in Starr County, Texas

Men	Aortic stiffness^a^		LV mass/height^2.7a^				Endothelial dysfunction^a^		Sleep apnea^a^		
	n	PWV ≥ 12 m/s %	n	Mild 49–55 %	Moderate 56–63 %	Severe ≥64 %	n	RHI < 1.67 %	N	Moderate %	Severe %
30–39	24	2.1	37	4.8	4.8	1.2	39	54.1	44	15.8	16.7
40–49	53	8.3	59	11.9	3.2	1.8	64	43.1	64	21.0	21.1
50–59	67	31.7	66	23.5	8.8	6.5	72	52.4	81	27.3	33.0
60–69	41	54.7	38	10.5	8.2	3.5	52	56.2	52	31.3	9.5
70+	16	39.7	24	31.9	11.3	20.3	24	21.6	24	6.8	36.5
Totals	201	22.3^b^	224	15.3^b^	7.0^b^	5.7^b^	251	48.6^b^	265	22.4^b^	22.8^b^

Women	Aortic stiffness^a^		LV mass/height^2.7a^				Endothelial dysfunction^a^		Sleep apnea^a^		
	n	PWV ≥ 12 %	n	Mild 45–51 %	Moderate 52–58 %	Severe ≥59 %	n	RHI < 1.67 %	N	15 ≤ AHI < 30 %	AHI ≥ 30 or CPAP %
30–39	106	1.1	114	9.1	2.6	3.6	128	30.5	132	7.9	3.7
40–49	151	4.9	147	15.5	5.8	6.6	174	19.2	196	15.9	5.8
50–59	167	20.8	148	23.2	13.0	11.3	183	24.0	202	21.4	17.2
60–69	62	44.5	59	26.8	18.5	24.8	69	21.9	84	33.4	18.1
70+	41	65.4	41	48.5	6.7	20.2	48	19.1	65	31.6	17.9
Totals	527	16.0^b^	509	17.8^b^	8.2^b^	9.7^b^	602	23.6^b^	679	17.9^b^	10.9
p value		0.216		0.172	0.308	0.003		0.000		0.356	0.000
♂vs♀ and OR^c^		1.30		0.75	0.73	0.36		3.24		1.18	2.42

^a^Frequencies in those with and without type 2 diabetes were weighted according to the age- and sex-specific population distributions of diabetes and no diabetes

^b^Population frequency among those 20 and above adjusted to the 2010 Census by the direct method

^c^p value based on Mantel–Haenszel Chi square testing for difference by sex after controlling for age effects. OR is the Mantel–Haenszel adjusted odds ratio

The less commonly considered factors in Table 2 indicate a much more complex disease burden than the presence of diabetes, obesity and hypertension. The Mantel–Haenszel Chi squares indicate significant differences by sex for the most increased LV mass category, impaired endothelial function and severe sleep apnea (AHI ≥ 30) after controlling for age. It is likely that these difference are reflective of differential distributions of the array of factors and these will be examined in more detail below. Additional file 1: Tables S1 and S2 provide data equivalent to Table 2 for those without and with type 2 diabetes, respectively.

There were 705 individuals without diabetes during the first examination (2002–2006) that were reexamined in 2010–2014. From this group, we detected 107 incident cases of type 2 diabetes during the average 8.5 year follow-up period yielding an incidence of 107/5978 person years or an annual incidence of 1.79 % (95 % confidence interval = 1.48–2.16 %). The annual incidence in those with prediabetes at the first examination was 3.48 % (2.80–4.3 %), fivefold higher than the 0.67 % (0.45–1.00 %) in those with normal glucose tolerance. Although HbA1c was not available during the first examination, we note that three additional individuals would be classified as having diabetes based on their HbA1c values at the follow-up examination.

Strengths of associations among conditions are in Table 3 where the odds ratios and significance from logistic regression are presented. The conditions most commonly considered, type 2 diabetes, obesity and hypertension, all show strong associations with one another. The strongest associations were between obesity and sleep apnea category (odds ratio = 3.16, p < 0.001) and type 2 diabetes and hypertension (odds ratio = 3.60, p < 0.001). Type 2 diabetes is also significantly associated with aortic stiffness and LV mass indexed for height after adjusting for age, sex, obesity and hypertension. In addition to sleep apnea, obesity is strongly associated with LV mass indexed for height while hypertension shows significant associations with all four non-traditional risk factors. Within the non-traditional risk factors, any associations among them seem to be largely explained already by their associations with age, sex, diabetes, obesity and hypertension. The exception is that LV mass shows a significant association with sleep apnea (odds ratio = 1.31, p < 0.05).

**Table 3:** Logistic regression odds ratios documenting the significance of associations between axis measures with age, sex, diabetes, obesity and hypertension always included in the model except when one of those was the dependent variable

Independent	Dependent						
	Diabetes	Obesity BMI ≥ 30	Hypertension	Aortic stiffness PWV ≥ 12	LV mass/height^2.7^ moderate +	Endothelial dysfunction RHI < 1.67	Sleep apnea AHI ≥ 15
Age group	2.36***	0.80***	2.57***	2.36***	1.72***	0.97	1.32***
Sex	1.48*	0.72*	1.50*	1.79*	0.75	3.27***	2.03***
Diabetes		1.43***	2.25***	2.79***	1.44*	1.24	1.34**
Obesity	1.28*		1.79***	0.95	3.96***	0.95	3.16***
Hypertension	3.60***	2.20***		4.21***	1.47	0.60**	1.92***
Aortic Stiffness	3.86***	0.86	4.01***		1.19	0.59	1.12
LV mass/height^2.7^	1.30**	2.24***	1.26*	1.12		0.95	1.34**
Endothelial dysfunction	1.28	1.02	0.60**	0.57	1.14		1.10
Sleep apnea	1.15	2.52***	1.31**	1.11	1.31*	0.99	

As independent variables, obesity uses three categorizations (normal, overweight and obese), diabetes uses three (normal glucose tolerance, prediabetes and diabetes) while LV mass and sleep apnea use four categorizations (none, mild, moderate and severe). As dependent variables, they are all dichotomized

* p ≤ 0.05

** p ≤ 0.01

*** p ≤ 0.001

Interviewer obtained data identified 138 individuals with prevalent cardiovascular disease based on a history of heart attack, angina, bypass surgery or stents. All but 17 of these individuals were in the group with diabetes who were significantly older than those without diabetes. Logistic regression was performed similarly to that in Table 3 with age, obesity and hypertension forced in a model to predict cardiovascular disease history and each non-traditional factor considered one at a time in analyses limited to those with type 2 diabetes. Of the non-traditional factors, LV mass showed a significant association with heart disease history after adjusting for the other factors (odds ratio = 1.44, p < 0.05) while the odds ratios for elevated pulse wave velocity (1.77), endothelial function (0.93), and sleep apnea (1.14) were not significantly different from 1. The lack of significance for pulse wave velocity was driven by the inclusion of age in the model.

**Fig. 2:**
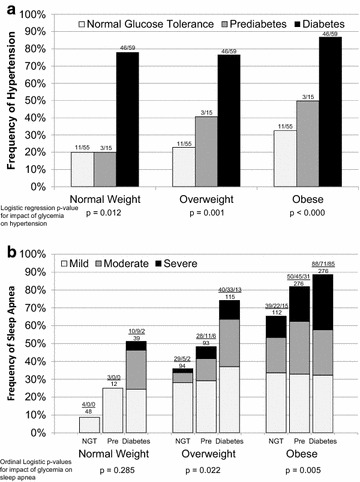
**a** The association between glycemic state and hypertension stratified by obesity among Mexican Americans in Starr County, Texas demonstrating a driving role of overt diabetes. Above *each bar* are the numbers of individuals hypertensive over the number of total individuals in said glycemia by weight category. p values are those obtained via logistic regression testing the hypothesis that glycemia (normal, prediabetes or diabetes) is a significant predictor of hypertension within each weight strata while adjusting for age and sex. **b** The association between moderate or more severe sleep apnea (AHI ≥ 15) and glycemic state stratified by obesity among Mexican Americans in Starr County, Texas (*NGT* normal glucose tolerance, *Pre* prediabetes, *Diabetes* type 2 diabetes). Above *each bar* are the numbers of individuals in each sleep apnea category over the number of total individuals in said glycemia by weight strata. p values are those obtained from ordinal logistic regression testing the hypothesis that glycemia is a significant predictor of sleep apnea (as an ordinal variable of none, mild, moderate and severe) within each weight strata while adjusting for age and sex

There is no one factor that seems to drive all the other factors. The relationship between diabetes and hypertension is an excellent case in point. Further examination of this relationship (Fig. 2a) shows that while obesity has a significant impact on hypertension, diabetes appears to be the real driving factor. Once diabetes is present, so too is hypertension in more than three quarters of individuals, irrespective of obesity status. This is not to say there is no association with obesity. As seen in Fig. 2a, there is a systematic increase in hypertension moving from normal weight to overweight to obese in those with normal glucose tolerance. This pattern is more pronounced in those classified with prediabetes, but the pattern appears essentially lost in those with diabetes.

Sleep apnea, on the other hand, appears driven more by obesity with a smaller contribution from diabetes (Fig. 2b). In Fig. 2b, there is a general increase in sleep apnea prevalence (at any threshold) across the obesity classifications that is most pronounced for moderate to severe sleep apnea. There is a smaller contribution due to diabetes that appears to interact with obesity (z-score = −2.12, p = 0.034 from ordinal logistic regression after adjusting for age and sex) with the clearest patterns in those classified as overweight or obese. The lack of such a pattern in the normal weight group likely reflects the much smaller sample sizes in the normal weight groups in the final sample. It is noteworthy that of the 155 individuals classified with severe sleep apnea, only 18 reported regular or intermittent use of CPAP.

**Fig. 3:**
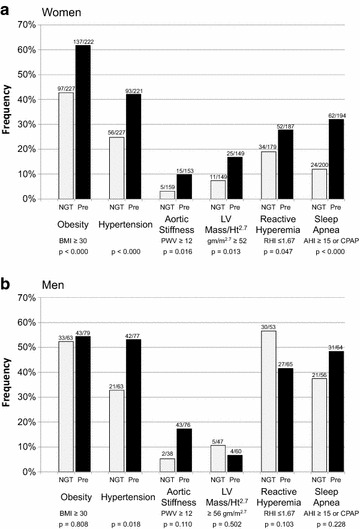
The impact of prediabetes on obesity, hypertension, aortic stiffness, left ventricular hypertrophy (moderate plus severe indexed by height^2.7^), impaired endothelial function and sleep apnea (moderate plus severe) among Mexican American women (**a**) and men (**b**) in Starr County, Texas. Prediabetes is based on a fasting blood glucose (100–125 mg/dl) or 2-h post-load glucose (140–199 mg/dl) without consideration of HbA1c. p values are those obtained from Chi square statistics (or Fisher’s exact test when cell size were five or less) testing the independence of prediabetes and the respective risk factor categorization

All indications are that these conditions and risk factors are developing early and in the absence of overt diabetes. Illustrated in Fig. 3a, b for men and women, respectively, are the relationship between prediabetes, defined by either fasting or 2-h post-load glucose, and obesity, hypertension, aortic stiffness, left ventricular hypertrophy, endothelial dysfunction and sleep apnea. In every instance except reactive hyperemia and LV mass in men, prediabetes is associated with a more untoward risk profile. Logistic regression analyses show that prediabetes is a significant predictor of obesity, hypertension, aortic stiffness, and sleep apnea after adjusting for age and sex. Frequencies among men and women in Fig. 3 are only significantly different for reactive hyperemia and sleep apnea and confirm what has been reported for them in other studies ([41] and [8], respectively).

## Discussion

The picture that emerges for this set of untoward health conditions is one of a large population burden coupled with complex interrelationships among factors. Tables 1 and 2 show the separate burdens among Mexican Americans, but do not capture the overall impact of this constellation of diseases and risk factors. In the full sample of 1200, 88 % have one or more of these conditions, 70 % have two or more, 50 % have three or more and 26 % have four or more. Excluding those with diabetes, 76, 43 and 20 % of the remainder have one, two, three or more of the other conditions, respectively. Furthermore, if we only consider the non-traditional risk factors of aortic stiffness, LV mass, impaired reactive hyperemia, and sleep apnea, 74 % of those with type 2 diabetes have one or more untoward values for these conditions while 58 % of those with prediabetes and 37 % of those without diabetes are so classified. Among those under age 50, these frequencies are 58, 51 and 31 %, respectively, indicating that the development of these often occurs before the full or even earliest metabolic consequences of diabetes are seen as we know that many of these younger adults will go on to develop diabetes. With such high frequencies across the spectrum, those individuals who have yet to develop any axis condition may represent a group that would be particularly fruitful for investigation as to why they may have some degree of protection.

It is clear that the conditions typically associated with diabetes already manifest changes in the prediabetes state as do the newer measures. Perhaps equally striking is the high burden in those with normal glucose tolerance. This would seem to push back the metabolic changes to being either precursors of the changes leading to compromised glucose metabolism or concomitant with other physiologic changes wherein glucose metabolism is only one of the manifestations.

With regard to classification of prediabetes we used the more conservative definition based on fasting and 2-h post-load glucose to maintain consistency with classifications from our earlier studies. A total of 304 individuals were classified as having prediabetes at the most recent examination based on fasting and 2-h post-load glucose. Considering HbA1c criteria [34] leads to an additional 47 individuals classified with prediabetes. Interestingly, only 12.3 % of those with prediabetes met the fasting, 2-h and HbA1c criteria. Furthermore, had we only used fasting glucose criteria, 36 % of those with prediabetes based on current criteria would have been missed. Utilizing fasting and 2-h post-load glucose misses 13 % while fasting glucose and HbA1c combined miss 16 % albeit they miss different individuals. The overlap of criteria are illustrated in Additional file 2: Figure S1. Adding the HbA1c classified individuals had minimal effect on the magnitude of associations illustrated in Fig. 2a, b. Additional file 3: Figure S2a, b are equivalent to Fig. 2a, b with the exception that HbA1c is considered in the definition of prediabetes.

The Mexican American population of Starr County has undergone rapid changes in the epidemiology and impact of type 2 diabetes, obesity, and hypertension when compared to results we reported two and three decades ago [42–44]. It appears that type 2 diabetes and obesity are on approximate 20 year doubling times. The disproportionate impact of type 2 diabetes, obesity and hypertension on the Mexican American population has been well documented, but there is little with regard to the other non-traditional risk factors documented here in the general or other Hispanic populations. Even within Hispanic groups reported in the Hispanic Community Health Study/Study of Latinos [8], these sleep apnea results indicate an elevated burden among Mexican Americans in the socioeconomically disadvantaged border community of Starr County. Some of these differences are likely explained by differences in sampling, equipment, and age structures. Even so, these studies demonstrate a significant, largely unrecognized and untreated burden of sleep apnea in the Mexican American population with consequences for other health outcomes. It is striking that 88.4 % of those who are obese and have diabetes have at least mild sleep apnea. These results are similar to the 86.6 % frequency of mild or worse sleep apnea in the Sleep AHEAD Study [45]. As in that study, nearly all of the sleep apnea among obese individuals with diabetes in Starr County is unrecognized. Our study and the Sleep AHEAD study make a compelling case for the need to consider sleep apnea in the routine assessment and treatment of obese individuals with type 2 diabetes.

## Conclusions

The full axis of untoward conditions is not only elevated in Starr County, but there are significant and complex associations among them. The patterns are consistent with a genesis well before overt disease classifications are recognized. This is most clear for prediabetes where there is an increasing appreciation for the need to intervene as a prevention and control strategy for coronary artery disease [46] and other conditions [47]. Whether these associations are mediated by common underlying factors or whether there exist multiple mechanisms remains to be seen. Answers will have significant public health and translational implications.

## Additional files

10.1186/s12933-016-0405-6 The frequencies by age and sex of aortic stiffness, LV mass, endothelial dysfunction and sleep apnea for those without type 2 diabetes (**Table S1**) and those with type 2 diabetes (**Table S2**).
10.1186/s12933-016-0405-6 Overlap of prediabetes classifications based on fasting blood glucose (100–125 mg/dl), 2-h post-load glucose (140–199 mg/dl and HbA1c (5.7–6.4 %) among Mexican Americans without diabetes in Starr County, Texas.
10.1186/s12933-016-0405-6 The impact of prediabetes on obesity, hypertension, aortic stiffness, left ventricular hypertrophy (moderate plus severe indexed by height^2.7^), impaired endothelial function and sleep apnea (moderate plus severe) among Mexican American women (2a) and men (2b) in Starr County, Texas. Prediabetes based on fasting blood glucose (100–125 mg/dl) or 2-h post-load glucose (140–199 mg/dl) or HbA1c (5.7–6.4 %). P values are those obtained from Chi square statistics (or Fisher’s Exact Test when cell size were five or less) testing the independence of prediabetes and the respective risk factor categorization.

## Abbreviations

AHI: apnea hypopnea index
BMI: body mass index
RHI: reactive hyperemia index
LV: left ventricular

## References

[1] Seetho IW, Parker RJ, Craig S, Duffy N, Hardy KJ, Wilding PH (2014). Obstructive sleep apnea is associated with increased arterial stiffness in severe obesity. J Sleep Res.

[2] Greve SV, Blicher MK, Blyme A, Sehestedt T, Hansen TW, Rassmusen S (2014). Association between albuminuria, atherosclerotic plaques, elevated pulse wave velocity, age, risk category and prognosis is apparently healthy individuals. J Hypertens.

[3] Osonoi Y, Mita T, Osonoi T, Saito M, Tamasawa A, Nakayama S (2015). Poor sleep quality is associated with increased arterial stiffness in Japanese patients with type 2 diabetes mellitus. BMC Endocr Disord.

[4] Lee JY, Ryu S, Lee SH, Kim BJ, Kim BS, Kang JH (2015). Association between brachial-ankle pulse wave velocity and progression of coronary artery calcium: a prospective cohort study. Cardiovasc Diabetol.

[5] Koren D, Chirinos JA, Katz LEL, Mohler ER, Gallagher PR, Mitchell GF (2015). Interrelationships between obesity, obstructive sleep apnea syndrome and cardiovascular risk in obese adolescents. Int J Obes.

[6] Daviglus ML, Talavera GA, Aviles-Santa ML, Allison M, Cai J, Criqui MH (2012). Prevalence of major cardiovascular risk factors and cardiovascular diseases among Hispanic/Latino individuals of diverse backgrounds in the United States. JAMA.

[7] Sorlie PD, Allison MA, Aviles-Santa M, Cai J, Daviglus ML, Howard AG (2014). Prevalence of hypertension, awareness, treatment, and control in the Hispanic Community Health Study/Study of Latinos. Am J Hypertens.

[8] Redline S, Sotres-Alvarez D, Loredo J, Hall M, Patel SR, Ramos A (2014). Sleep-disordered breathing in Hispanic/Latino individuals of diverse backgrounds. The Hispanic Community Health Study/Study of Latinos. Am J Respir Crit Care Med.

[9] Nichols WW, Denardo SJ, Wilkinson IB, McEniery CM, Cockcroft J, O’Rourke MF (2008). Effects of arterial stiffness, pulse wave velocity, and wave reflection on the central aortic pressure wave form. J Clin Hypertens.

[10] Nemes A, Geleijnse ML, Forster T, Soliman OI, Ten Cate FJ, Csanady M (2008). Echocardiographic evaluation and clinical implications of aortic stiffness and coronary flow reserve and their relation. Clin Cardiol.

[11] Yasmin, O’Shaughnessy KM (2008). Genetics of arterial structure and function: towards new biomarkers for aortic stiffness. Clin Sci.

[12] Sharman JE, Haluska BA, Fang ZY, Prins JB, Marwick TH (2007). Association of arterial wave properties and diastolic dysfunction in patients with type 2 diabetes mellitus. Am J Cardiol.

[13] Lang RM, Bierig M, Devereux RB, Flachskampf FA, Foster E, Pellikka PA (2005). Recommendations for chamber quantification: a report from the American Society of Echocardiography’s Guidelines and Standards Committee and the Chamber Quantification Writing Group, developed in conjunction with the European Association of Echocardiography, a branch of the European Society of Cardiology. J Am Soc Echocardiogr.

[14] Lavie CJ, Patel DA, Milani RV, Ventura HO, Shah S, Gilland Y (2014). Impact of echocardiographic left ventricular geometry on clinical prognosis. Prog Cardiovasc Dis.

[15] Gottlieb DJ (2008). The Sleep Heart Health Study: a progress report. Curr Opin Pulm Med.

[16] Usui Y, Takata Y, Inoue Y, Tomiyama H, Kurohane S, Hashimura Y (2013). Severe obstructive sleep apnea impairs left ventricular diastolic function in non-obese men. Sleep Med.

[17] Correia MLG, Haynes WG (2007). Arterial compliance and endothelial function. Curr Diabetes Rep.

[18] Phillips C, Hedner J, Berend N, Grunstein R (2005). Diurnal and obstructive sleep apnea influences on arterial stiffness and central blood pressure in men. Sleep.

[19] Rodriguez CJ, Miyake Y, Grahame-Clarke C, Di Tullio MR, Sciacca RR, Boden-Albala B (2005). Relation of plasma glucose and endothelial function in a population-based multiethnic sample of subjects without diabetes mellitus. Am J Cardiol.

[20] Wajcberg E, Thoppil N, Patel S, Fernandez M, Hale D, DeFronzo R (2006). Comprehensive assessment of postischemic vascular reactivity in Hispanic children and adults with and without diabetes mellitus. Pediatr Diabetes.

[21] Nathanson D, Nystrom T (2009). Hypoglycemic pharmacologic treatment of type 2 diabetes: targeting the endothelium. Mol Cell Endocrinol.

[22] Nieto FJ, Herrington DM, Redline S, Benjamin EJ, Robbins JA (2003). Sleep apnea and markers of endothelial function in a large community sample of older adults. Am J Respir Crit Care Med.

[23] Atkeson A, Jelic S (2008). Mechanisms of endothelial dysfunction in obstructive sleep apnea. Vasc Health Risk Manag.

[24] Quan SF, Howard BV, Iber C, Kiley JP, Nieto FJ, O’Connor GT (1997). The Sleep Heart Health Study: design, rationale, and methods. Sleep.

[25] Shaw JE, Punjabi NM, Wilding JP, Alberti KGMM, Zimmet PZ (2008). Sleep-disordered breathing and type 2 diabetes—a report from the International Diabetes Federation Taskforce on Epidemiology and Prevention. Diabetes Res Clin Pract.

[26] Punjabi NM, Caffo BS, Goodwin JL, Gottlieb DJ, Newman AB, O’Connor GT (2009). Sleep-disordered breathing and mortality: a prospective cohort study. PLoS Med.

[27] Below JE, Gamazon ER, Morrison JV, Konkashbaev A, Pluzhnikov A, McKeigue PM (2011). Genome-wide association and meta-analysis in populations from Starr County, Texas and Mexico City identify type 2 diabetes susceptibility loci and enrichment for eQTLs in top signals. Diabetologia.

[28] Aguilar D, Hallman DM, Piller LB, Klein BEK, Klein R, Devereux RB (2009). Adverse association between diabetic retinopathy and cardiac structure and function. Am Heart J.

[29] Ring M, Eriksson MJ, Zierath JR, Caidahl K (2014). Arterial stiffness estimation in healthy subjects: a validation of oscillometric (Arteriograph) and tonometric (SphygmoCor) techniques. Hypertens Res.

[30] Reisner Y, Lusky R, Shay-El Y, Schnall R, Herscovici S (2008). Reproducibility of endothelial function and arterial stiffness assessed using finger peripheral arterial tonometry. Eur Heart J.

[31] Patvardhan EA, Heffernan KS, Ruan JM, Soffler MI, Karas RH, Kuvin JT (2010). Assessment of vascular endothelial function with peripheral arterial tonometry: information at your fingertips?. Cardiol Rev.

[32] Woo JS, Jang WS, Kim HS, Lee JH, Choi EY, Kim JB (2014). Comparison of peripheral arterial tonometry and flow-mediated vasodilation for assessment of the severity and complexity of coronary artery disease. Coron Artery Dis.

[33] Yalamanchali S, Farajian V, Hamilton C, Pott TR, Samuelson CG, Friedman M (2013). Diagnosis of obstructive sleep apnea by peripheral arterial tonometry: meta-analysis. JAMA Otolaryngol Head Neck Surg.

[34] American Diabetes Association (2015). Standards of medical care in diabetes—2015: 2. classification and diagnosis of diabetes. Diabetes Care.

[35] NIH. Clinical guidelines on the identification, evaluation, and treatment of overweight and obesity in adults: the evidence report. Washington, DC. 1998. (NIH publ. no. 98–4083). 9813653

[36] Giles TD, Berk BC, Black HR, Cohn JN, Kostis JB, Izzo JL (2005). Expanding the definition and classification of hypertension. J Clin Hypertens.

[37] The Reference Values for Arterial Stiffness Collaboration (2010). Determinants of pulse wave velocity in healthy people and in the presence of cardiovascular risk factors: ‘establishing normal reference values’. Eur Heart J.

[38] Syvanen K, Korhonen P, Partanen A, Aarnio P (2011). Endothelial function in a cardiovascular risk population with borderline ankle-brachial index. Vasc Health Risk Manag.

[39] Rothman KJ, Greenland S, Lash TL (2008). Modern epidemiology.

[40] Selvin S (2004). Statistical analysis of epidemiologic data.

[41] Carnovale V, Paradis ME, Gigleux I, Ramprasath VR, Couture P, Jones PJ (2013). Correlates of reactive hyperemic index on men and postmenopausal women. Vasc Med.

[42] Hanis CL, Ferrell RE, Barton SA, Aguilar L, Garza-Ibarra A, Tulloch BR (1983). Diabetes among Mexican-Americans in Starr County,Texas. Am J Epidemiol.

[43] Hanis CL, Hewett-Emmett D, Kubrusly LF, Maklad MN, Douglas TC, Mueller WH (1993). An ultrasound survey of gallbladder disease among Mexican Americans in Starr County, Texas: frequencies and risk factors. Ethn Dis.

[44] Hanis CL, Ferrell RE, Schull WJ (1985). Hypertension and sources of blood pressure variability among Mexican-Americans in Starr County, Texas. Int J Epidemiol.

[45] Foster GD, Sanders MH, Millman R, Zammit G, Borradaile KE, Newman AB (2009). Obstructive sleep apnea among obese patients with type 2 diabetes. Diabetes Care.

[46] Goldfine AB, Phua EJ, Abrahamson MJ (2014). Glycemic management in patients with coronary artery disease and prediabetes or type 2 diabetes mellitus. Circulation.

[47] Grundy SM (2012). Pre-diabetes, metabolic syndrome, and cardiovascular risk. J Am Coll Cardiol.

