# Supplementary material for: Beyond type 2 diabetes, obesity and hypertension: an axis including sleep apnea, left ventricular hypertrophy, endothelial dysfunction, and aortic stiffness among Mexican Americans in Starr County, Texas
Source: Cardiovasc Diabetol. 2016 Jun 8;15:86. doi: 10.1186/s12933-016-0405-6 (PMC4897940; doi:10.1186/s12933-016-0405-6)
Supplement: Supplementary file 1 — 10.1186/s12933-016-0405-6 The frequencies by age and sex of aortic stiffness, LV mass, endothelial dysfunction and sleep apnea for those without type 2 diabetes (Table S1) and those with type 2 diabetes (Table S2). [file 12933_2016_405_MOESM1_ESM.docx]

| Table S1. Aortic Stiffness, LV Mass, Endothelial Dysfunction and Sleep Apnea by Age and Sex Among Mexican Americans Without Type 2 Diabetes in Starr County, Texas. | | | | | | | | | | | |
| --- | --- | --- | --- | --- | --- | --- | --- | --- | --- | --- | --- |
|  | Aortic Stiffness | | LV Mass/Height^2.7^ | | | | Endothelial Dysfunction | | Sleep Apnea | | |
| Males | n | PWV ≥ 12  % | n | Mild  49-55  % | Moderate  56-63  % | Severe  ≥ 64  % | n | RHI < 1.67  % | n | 15 ≤ AHI <30  % | AHI ≥ 30 or CPAP  % |
| 30 – 39 | 17 | 0.0 | 25 | 4.0 | 4.0 | 0.0 | 28 | 57.1 | 32 | 15.2 | 12.1 |
| 40 – 49 | 34 | 5.9 | 41 | 12.2 | 2.4 | 2.4 | 45 | 40.0 | 43 | 23.3 | 18.6 |
| 50 – 59 | 33 | 21.2 | 38 | 21.1 | 7.9 | 7.9 | 39 | 51.3 | 40 | 27.5 | 30.0 |
| 60 – 69 | 5 | 40.0 | 2 | 0.0 | 0.0 | 0.0 | 5 | 60.0 | 3 | 33.3 | 0.0 |
| 70 + | 1 | 0.0 | 2 | 50.0 | 0.0 | 0.0 | 1 | 0.0 | 2 | 0.0 | 50.0 |
| Totals | 90 | 12.1 | 108 | 13.9 | 4.6 | 3.7 | 118 | 48.3 | 121 | 22.3 | 20.7 |
|  | | | | | | | | | | | |
| Females | n | PWV ≥ 12  % | n | Mild  45-51  % | Moderate  52-58  % | Severe  ≥ 59  % | n | RHI < 1.67  % | n | 15 ≤ AHI <30  % | AHI ≥ 30 or CPAP  % |
| 30 – 39 | 96 | 0.0 | 103 | 5.8 | 2.9 | 2.9 | 116 | 30.2 | 120 | 5.9 | 4.2 |
| 40 – 49 | 104 | 2.9 | 99 | 15.2 | 4.0 | 4.0 | 125 | 18.4 | 140 | 13.6 | 5.0 |
| 50 – 59 | 86 | 8.1 | 78 | 21.8 | 11.5 | 9.0 | 100 | 22.0 | 108 | 20.4 | 13.9 |
| 60 – 69 | 17 | 35.3 | 13 | 30.8 | 23.1 | 23.1 | 18 | 27.8 | 20 | 30.0 | 15.0 |
| 70 + | 9 | 44.4 | 4 | 75.0 | 0.0 | 0.0 | 7 | 14.3 | 6 | 33.3 | 0.0 |
| Totals | 311 | 6.4 | 297 | 15.2 | 6.4 | 5.7 | 366 | 23.5 | 393 | 14.2 | 7.6 |

| Table S2. Aortic Stiffness, LV Mass, Endothelial Dysfunction and Sleep Apnea by Age and Sex Among Mexican Americans With Type 2 Diabetes in Starr County, Texas. | | | | | | | | | | | |
| --- | --- | --- | --- | --- | --- | --- | --- | --- | --- | --- | --- |
|  | Aortic Stiffness | | LV Mass/Height^2.7^ | | | | Endothelial Dysfunction | | Sleep Apnea | | |
| Males | n | PWV ≥ 12  % | n | Mild  49-55  % | Moderate  56-63  % | Severe  ≥ 64  % | n | RHI < 1.67  % | n | 15 ≤ AHI <30  % | AHI ≥ 30  % |
| 30 – 39 | 7 | 12.5 | 12 | 8.3 | 8.3 | 8.3 | 11 | 36.4 | 12 | 16.7 | 41.7 |
| 40 – 49 | 19 | 15.8 | 18 | 11.1 | 5.6 | 0.0 | 19 | 52.6 | 21 | 14.3 | 28.6 |
| 50 – 59 | 34 | 52.9 | 28 | 28.6 | 10.7 | 3.6 | 33 | 54.5 | 41 | 26.8 | 39.0 |
| 60 – 69 | 36 | 75.0 | 36 | 25.0 | 19.4 | 8.3 | 47 | 51.1 | 49 | 28.6 | 22.4 |
| 70 + | 15 | 80.0 | 22 | 13.6 | 22.7 | 40.9 | 23 | 43.5 | 22 | 13.6 | 22.7 |
| Totals | 111 | 54.5 | 116 | 19.8 | 14.7 | 12.1 | 133 | 49.6 | 145 | 22.8 | 29.7 |
|  | | | | | | | | | | | |
| Females | N | PWV ≥ 12  % | n | Mild  45-51  % | Moderate  52-58  % | Severe  ≥ 59  % | n | RHI < 1.67  % | n | 15 ≤ AHI <30  % | AHI ≥ 30  % |
| 30 – 39 | 10 | 10.0 | 11 | 36.4 | 0.0 | 9.1 | 12 | 33.3 | 12 | 25.0 | 0.0 |
| 40 – 49 | 47 | 12.8 | 48 | 16.7 | 12.5 | 16.7 | 49 | 22.4 | 56 | 25.0 | 8.9 |
| 50 – 59 | 81 | 44.4 | 70 | 25.7 | 15.7 | 15.7 | 83 | 27.7 | 94 | 23.4 | 23.4 |
| 60 – 69 | 45 | 51.1 | 46 | 23.9 | 15.2 | 26.1 | 51 | 17.6 | 64 | 35.9 | 20.3 |
| 70 + | 32 | 78.1 | 37 | 32.4 | 10.8 | 32.4 | 41 | 22.0 | 59 | 30.5 | 28.8 |
| Totals | 215 | 42.3 | 212 | 25.0 | 13.2 | 20.8 | 236 | 23.7 | 285 | 28.1 | 20.0 |
