# Supplementary figures and images for: Beyond type 2 diabetes, obesity and hypertension: an axis including sleep apnea, left ventricular hypertrophy, endothelial dysfunction, and aortic stiffness among Mexican Americans in Starr County, Texas
Source: Cardiovasc Diabetol. 2016 Jun 8;15:86. doi: 10.1186/s12933-016-0405-6 (PMC4897940; doi:10.1186/s12933-016-0405-6)

## Slide 1
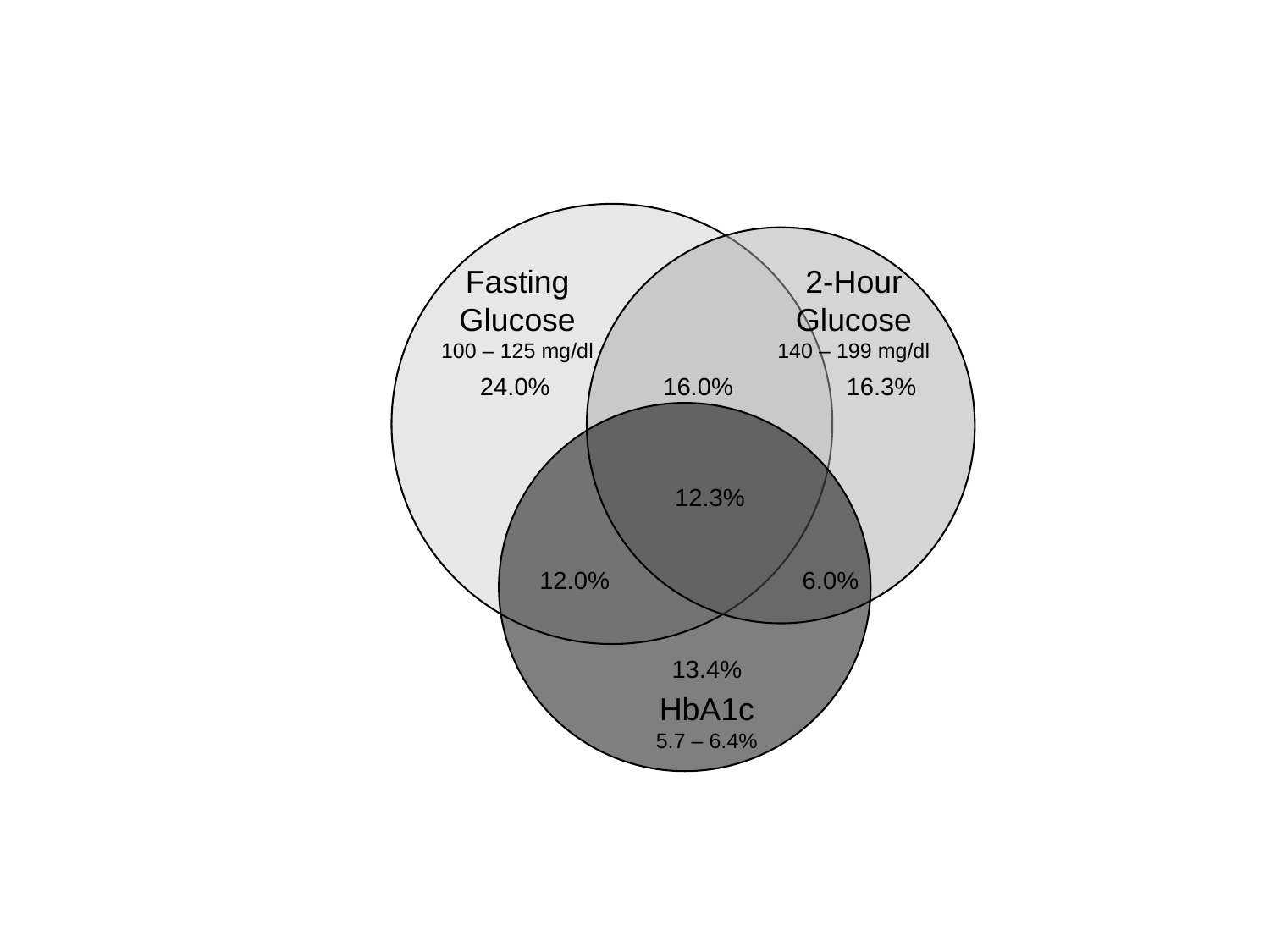

Fasting
Glucose
100 – 125 mg/dl
2-Hour
Glucose
140 – 199 mg/dl
24.0%
16.3%
16.0%
12.3%
12.0%
6.0%
13.4%
HbA1c
5.7 – 6.4%

Supplement: Supplementary file 2 — 10.1186/s12933-016-0405-6 Overlap of prediabetes classifications based on fasting blood glucose (100–125 mg/dl), 2-h post-load glucose (140–199 mg/dl and HbA1c (5.7–6.4 %) among Mexican Americans without diabetes in Starr County, Texas. [file 12933_2016_405_MOESM2_ESM.pptx]
