# Supplementary material for: Beyond type 2 diabetes, obesity and hypertension: an axis including sleep apnea, left ventricular hypertrophy, endothelial dysfunction, and aortic stiffness among Mexican Americans in Starr County, Texas
Source: Cardiovasc Diabetol. 2016 Jun 8;15:86. doi: 10.1186/s12933-016-0405-6 (PMC4897940; doi:10.1186/s12933-016-0405-6)
Supplement: Supplementary file 3 — 10.1186/s12933-016-0405-6 The impact of prediabetes on obesity, hypertension, aortic stiffness, left ventricular hypertrophy (moderate plus severe indexed by height2.7), impaired endothelial function and sleep apnea (moderate plus severe) among Mexican American women (2a) and men (2b) in Starr County, Texas. Prediabetes based on fasting blood glucose (100–125 mg/dl) or 2-h post-load glucose (140–199 mg/dl) or HbA1c (5.7–6.4 %). P values are those obtained from Chi square statistics (or Fisher’s Exact Test when cell size were five or less) testing the independence of prediabetes and the respective risk factor categorization. [file 12933_2016_405_MOESM3_ESM.pptx]

## Slide 1
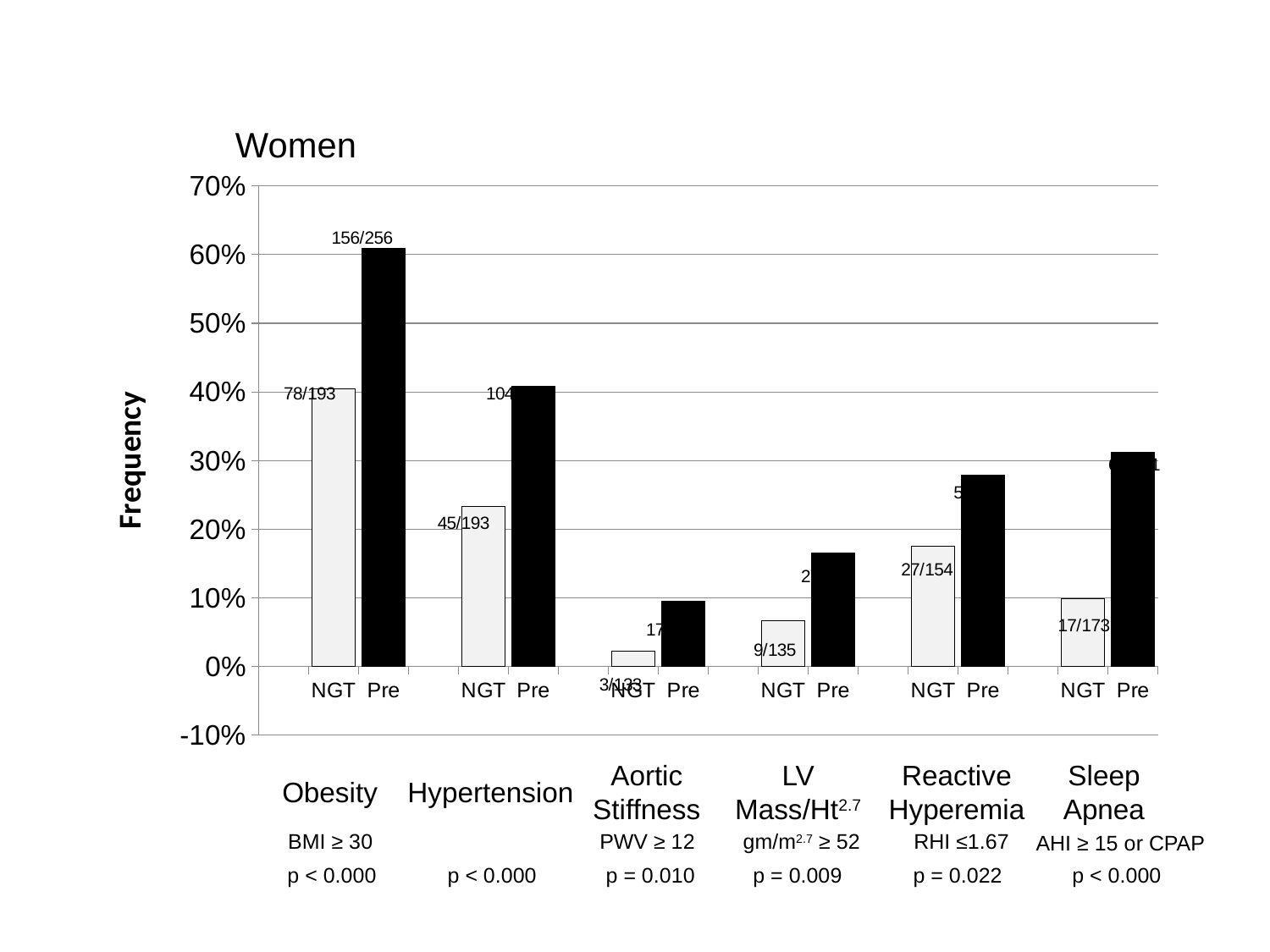

Women
### Chart
| Category | Column1 |
|---|---|
| | None |
| NGT | 0.4041 |
| Pre | 0.6094 |
| | None |
| NGT | 0.2332 |
| Pre | 0.40780000000000005 |
| | None |
| NGT | 0.022600000000000002 |
| Pre | 0.09500000000000001 |
| | None |
| NGT | 0.06670000000000001 |
| Pre | 0.16560000000000002 |
| | None |
| NGT | 0.1753 |
| Pre | 0.27830000000000005 |
| | None |
| NGT | 0.09830000000000004 |
| Pre | 0.31220000000000003 |Aortic
Stiffness
LV
Mass/Ht2.7
Reactive
Hyperemia
Sleep
Apnea
Obesity
Hypertension
BMI ≥ 30
PWV ≥ 12
gm/m2.7 ≥ 52
RHI ≤1.67
AHI ≥ 15 or CPAP
p < 0.000
p < 0.000
p = 0.010
p = 0.009
p = 0.022
p < 0.000

## Slide 2
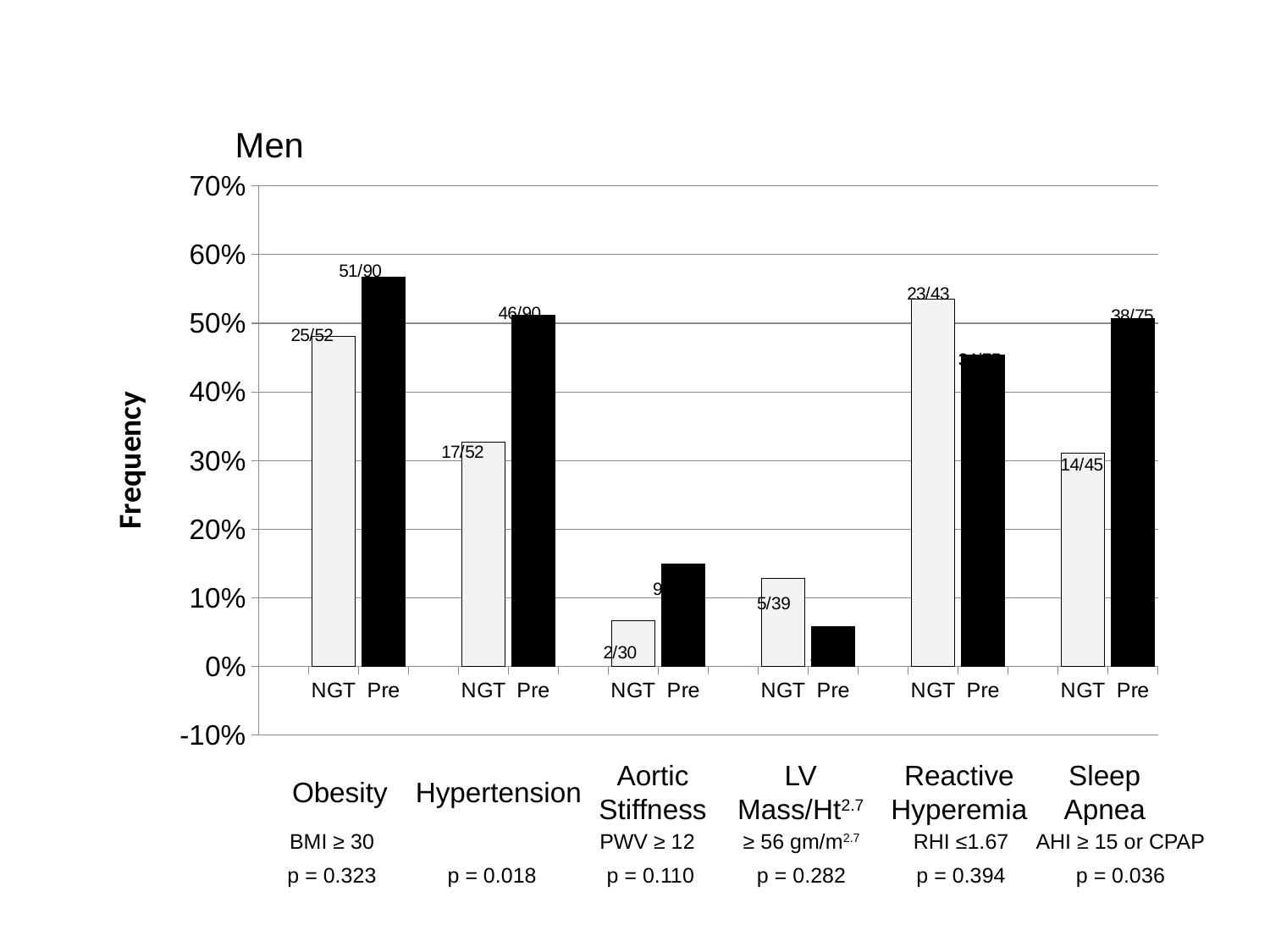

Men
### Chart
| Category | Column1 |
|---|---|
| | None |
| NGT | 0.48080000000000017 |
| Pre | 0.5667000000000003 |
| | None |
| NGT | 0.32690000000000025 |
| Pre | 0.5111 |
| | None |
| NGT | 0.0667 |
| Pre | 0.15000000000000008 |
| | None |
| NGT | 0.1282 |
| Pre | 0.05880000000000001 |
| | None |
| NGT | 0.5349 |
| Pre | 0.4533 |
| | None |
| NGT | 0.3111000000000002 |
| Pre | 0.5067 |Aortic
Stiffness
LV
Mass/Ht2.7
Reactive
Hyperemia
Sleep
Apnea
Obesity
Hypertension
BMI ≥ 30
PWV ≥ 12
≥ 56 gm/m2.7
RHI ≤1.67
AHI ≥ 15 or CPAP
p = 0.323
p = 0.018
p = 0.110
p = 0.282
p = 0.394
p = 0.036
